# Will prior health insurance authorization for medications continue to hinder hepatitis C treatment delivery in the United States? Perspectives from hepatitis C treatment providers in a large urban healthcare system

**DOI:** 10.1371/journal.pone.0241615

**Published:** 2020-11-04

**Authors:** Marjan Javanbakht, Roxanne Archer, Jeffrey Klausner

**Affiliations:** 1 Department of Epidemiology, Fielding School of Public Health, University of California, Los Angeles, California, United States of America; 2 Division of Infectious Diseases, Department of Medicine, David Geffen School of Medicine at University of California, Los Angeles, California, United States of America; Middle East Liver Diseases (MELD) Center, ISLAMIC REPUBLIC OF IRAN

## Abstract

**Background:**

The recent introduction of direct acting antivirals for the treatment of hepatitis C virus (HCV) has dramatically improved treatment options for HCV infected patients. However, in the United States (US) treatment uptake has been low and time to initiation of therapy has been long. We sought to examine provider perspectives of facilitators and barriers to HCV treatment delivery.

**Methods:**

From June to August 2019, we conducted in-depth, semi-structured interviews with medical staff providing HCV care as part of a university medical center in Los Angeles, CA. In order to understand the HCV treatment process, we interviewed key staff members providing care to the majority of HCV patients seeking care at the university medical center, including hepatologists and infectious disease specialists as well as key nursing and pharmacy staff. The interviews focused on workload and activities required for HCV treatment initiation for non-cirrhotic, treatment naïve patients.

**Results:**

Providers noted that successful HCV treatment delivery was reliant on a care model involving close collaboration between a team of providers, in particular requiring a highly coordinated effort between dedicated nursing and pharmacy staff. The HCV care team overwhelmingly reported that the process of insurance authorization was the greatest obstacle delaying treatment initiation and noted that very few patient level factors served as a barrier to treatment uptake.

**Conclusions:**

In the US, prior authorization for HCV treatment is a requirement for most public and private insurance plans. In an era with access to therapies that allow for a cure—and until revocation of prior authorization for HCV treatment is a reality—implementing strategies that can expedite authorization to accelerate treatment access are critical. Not only will this benefit patients, but it has the potential to help expand treatment to settings that are otherwise too resource strained to successfully deliver HCV care.

## Introduction

It is estimated that 2.4 million people in the United States (US) are infected with hepatitis C virus (HCV), making it one of the most common blood borne infections in the US [1, 2]. Transmission of HCV occurs through contact with infected body fluids—in particular blood—making injection drug use one of the most common risk factors for HCV acquisition in the US [1, 3–5]. The prevalence of HCV infections is highest among men, African Americans/blacks, followed by Caucasians/whites, as well as persons in the birth cohort born between 1945 and 1965 –a generational cohort known as ‘baby boomers’ [1]. Recent surveillance data suggest that not only are incident infections increasing but the number of cases among persons born after 1965 have been increasing with the highest incidence among those 20–29 years of age, followed by those 30–39 years [1]. In fact, one study estimates that there has been a near doubling of acute HCV infections among women with live births between 2009 and 2014 [6].

Acute HCV infection is typically asymptomatic or mild [7, 8]. However, chronic infections with HCV—which is estimated to occur in at least half of all cases—are the leading cause of liver disease including, fibrosis, cirrhosis, hepatocellular carcinoma, and liver-related deaths [9–14]. The introduction of direct acting antivirals (DAA) in 2011 has dramatically changed the treatment trajectory for HCV infected patients [15]. Compared to interferon-based regimen, DAAs are better tolerated, have a shorter duration of treatment, and are more effective allowing for sustained virologic response (i.e., cure) in almost all cases [16, 17]. These new therapies have greatly increased the number of patients that can be successfully treated. Furthermore, the introduction of increasingly shorter regimen has the potential to improve treatment uptake and adherence while reducing both clinical and patient burden associated with treatment.

Treatment initiation following HCV diagnosis has been low with a number of studies indicating that less than a third of patients initiated HCV treatment with DAA-containing regimens [18–20]. Following diagnosis, barriers to HCV treatment initiation have included the presence of comorbidities such as substance use and psychiatric disorders, medical ineligibilities such as liver disease too advanced to tolerate treatment, as well as patient attitudes including low confidence in the effectiveness of treatment and fear of side effects [18–23]. Within the US context, economic considerations have also played a significant role. For those who lack health insurance, the out-of-pocket treatment cost (i.e., self-pay) is prohibitive for most, with prices ranging upwards of $100,000 (US dollars) for completion of treatment [24, 25]. Those with health insurance require approval by their insurance provider in order to receive coverage of HCV treatment costs. The approval process also known as ‘pre-authorization’ or ‘prior-authorization’ is the process by which health insurance providers review the medical necessity of a treatment and therefore allow for coverage of treatment costs.

Current treatment guidelines in the US recommend eight week treatment with Glecaprevir/pibrentasvir or twelve week treatment with Sofosbuvir/velpatasvir for HCV genotype-1, treatment naïve, non-cirrhotic patients, with both regimen yielding comparable treatment outcomes [14, 18, 26, 27]. Beyond low treatment initiation rates, time to treatment initiation has also been long. A recent study of over 8,000 HCV infected patients in the United States, found that less than 10% initiated treatment with DAAs within the two year study period and the median time to treatment initiation was 300 days [18]. Patient level factors positively associated with treatment initiation included prior HCV treatment, cirrhosis, and history of liver transplant while negative factors included a history of substance use and being uninsured (i.e., self-pay) or having insurance through federal medical assistance programs rather than private health insurance [18]. Furthermore, it’s estimated that less than half of patients eligible for shorter duration treatment received it [26]. The simplified, shorter duration treatment has the potential to expand resources available for treatment delivery and increase the number of HCV infected persons treated, which closely aligns with national and international targets to increase HCV treatment and reduce the number of chronic HCV infections [28, 29]. Understanding the clinical and structural factors associated with treatment initiation and the choice of treatment may allow for increased uptake of treatment overall, as well as expanded use of shorter treatment regimen. Our objective was to explore provider perspectives on the primary barriers and facilitators of HCV treatment delivery as well as factors influencing whether eligible patients receive eight or twelve week treatment regimen.

## Methods

The study was approved by the institutional review board at the University of California, Los Angeles. The need for consent was waived by the ethics committee. From June to August 2019, we conducted in-depth, semi-structured interviews with medical staff providing HCV care as part of a university medical center and the affiliated primary care and specialty clinics in Los Angeles, CA. The health care system which serves as the setting for this study consists of approximately sixty outpatient care clinics and two hospitals and serves an estimated 500,000 patients per year (unpublished health system data). The majority of patients receive HCV care through specialty care clinics (hepatologist) with a lesser number receiving care through infectious disease specialists. Within the system, delivery of HCV care is dependent both on providers (physician and physician extenders) and other medical staff, with heavy reliance on medical assistants, nursing staff, as well as pharmacy staff. Given this team approach, the medical staff play a critical role in the HCV continuum of care including patient intake, assessment of treatment readiness, uptake and adherence issues, as well as follow-up to ensure that treatment is completed.

In order to understand the full spectrum of the HCV treatment process, we interviewed all key staff members (n = 6) providing care to the majority of HCV patients within this health system, including two hepatologists, one infectious disease specialist, one nurse who serves as the primary HCV care coordinator in the hepatology clinic, and one clinical pharmacist who oversees HCV treatment coordination within the pharmacy. The nurse and pharmacist were the only two staff members within the system to serve as HCV care coordinators, while the hepatology clinic is staffed by six physicians. The two physicians interviewed as part of this study were chosen given their specialization in HCV care and their higher HCV patient load. In addition, while non-specialist physicians (e.g., family medicine physicians) also provide HCV care within this system, the number of HCV patients any given provider serves is significantly limited by comparison and we were interested in understanding barriers and facilitators to care in a setting specifically focused on HCV, potentially removing barriers that result from lack of specialization in delivering HCV care.

**Fig 1:**
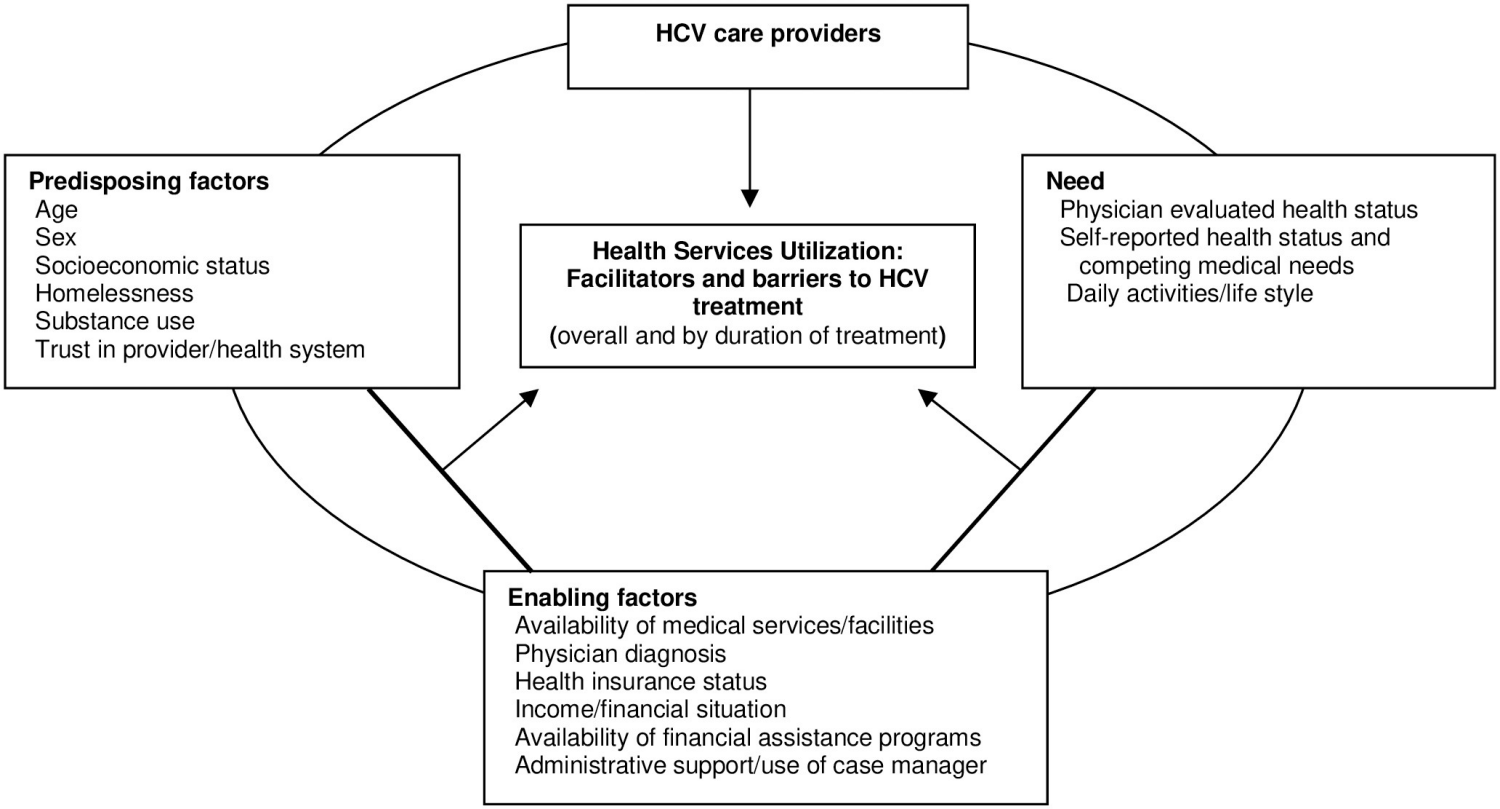
Conceptual model for HCV care providers perspectives on facilitators and barriers of HCV treatment as influenced by the three domains of the Andersen behavioral model.

The 30-minute interviews were conducted in person, one-on-one, and used a semi-structured, open-ended script. The interview guide was informed by Andersen’s behavioral model of health services utilization [30]. The model considers three main factors in individuals’ utilization of health care services including the predisposition to use health care services (“predisposing factors”), factors which enable or impede health care use (“enabling factors”), and an individuals need for care (“need”). Predisposing factors includes a consideration of socio-demographic characteristics such as age, gender, and socioeconomic status, as well as health beliefs. Enabling factors include availability and access to resources (e.g., availability of medical facilities and economic means to access these resources). Needs includes both perceived need and evaluated need based on medical judgment. The conceptual framework as well as the specific factors considered within each of the domains relevant to this study are depicted in Fig 1.

Interviews followed established qualitative interview methodology combining open-ended questions as well as structured probes to explore factors in each of the three domains within the Andersen behavioral model [31]. Each interview began with general questions about the clinic and the participant’s role in the clinic, with the remainder of the interview focusing on experiences with HCV care delivery. Interview questions followed themes designed to understand barriers and facilitators of HCV treatment delivery with a focus on differences in structural, clinical, and patient related factors overall as well as by treatment duration (eight vs. twelve week). The interviews allowed for a more in depth exploration of the following topics as they related to HCV treatment for non-cirrhotic, treatment naïve patients: (1) workload and activities performed in order to ensure medication initiation; (2) case management including laboratory testing, follow-up, and adherence checks conducted during the course of treatment; and (3) factors that go into deciding whether to start a patient on an 8-week vs. 12-week regimen. A listing of the question used in the interview guide are provided in Table 1. Following the interviews, the response text were searched, labeled, extracted, and categorized for each topic of interest using content analysis. The themes were then analyzed for content pertaining to our research objectives. The interviews were independently reviewed by two study investigators to resolve any discrepancies. The study was approved by the Institutional Review Board at the University of California Los Angeles.

**Table 1:** Interview guide for provider perceptions of facilitators and barriers of HCV treatment.

The interview was conducted in such a way as to enable and encourage the respondent to freely express their experience and narrative around the question at hand.		
	**Question and probe**	
1.	Could describe your role in the clinic?	
2.	What factors go into deciding whether to start a non-cirrhotic patient on treatment?	
	a.	How does this differ for 8-week vs. 12-week regimen?
	b.	Are there additional tests or visits conducted beyond what would be done if a 12-week regimen was being used?
		Could you please describe this.
	c.	Are there any patient preferences that play a role in deciding between regimen types?
3.	What types of activities occur and what type of tasks do staff perform for to get patients started on HCV treatment?	
	a.	What is the medication approval/ pre-authorization process like?
	b.	Does this differ by regimen?
4.	After a prescription is ordered, what type of further laboratory or clinical follow-up is conducted or scheduled?	
	a.	What are the differences for an 8-week vs. 12-week regimen?
5.	What type and frequency of follow-up or adherence checks or outreach occur by clinic staff during treatment?	
	a.	What are the differences for an 8-week vs. 12-week regimen?
6.	When is the post treatment follow-up scheduled?	
	a.	What is the process for scheduling post-treatment SVR?

The interview concluded with a summary by the interviewer in order to verify that the content was understood correctly

## Results

### Process for HCV treatment delivery

We found that the process of HCV treatment initiation involves a highly coordinated effort between dedicated nursing and pharmacy staff. Once a physician has determined treatment is indicated, the nursing coordinator starts the process by ensuring that all laboratory testing and assessments have been completed. A common missing laboratory component, which is a requirement for insurance approval is HCV genotype testing. Consequently, it is not unusual for the nurse to initiate follow-up testing and patient coordination to ensure that all necessary lab results for insurance authorization are in place.

Once all the requisite material are ready, the nurse coordinator prepares a pre-authorization packet which consists of all relevant paperwork including findings from the clinical evaluation, laboratory results, and administrative requirements necessary for insurance authorization. The pre-authorization packet is sent to a dedicated in-house pharmacist who serves as the pharmacy ‘case manager’ in order to usher the approval process through the system. The steps can include verifying coverage, outreach to patient assistance programs to mitigate cost of treatment including those whose health insurance may not cover the entirety of the cost, as well as follow-up calls to ensure that medications have been ordered and shipped. The pharmacist estimates that each case requires several phone calls and can take several hours to manage these efforts. Both the nurse and the pharmacist noted that there were no differences in the process or time required to submit insurance authorization paper-work for either the eight week or twelve week regimen.

Once insurance coverage has been authorized, the nurse coordinates with the patient to ensure medication pick-up/delivery with additional calls to verify treatment start date. This in turn will allow the nurse coordinator to schedule a follow-up visit after four weeks of treatment for laboratory testing.

Beyond coordinating the start of treatment, the nurse remains available throughout the course of treatment to address any patient concerns and continues to manage the process by tracking shipment dates for refills, ensuring patients are available to receive refills, and coordinating and scheduling follow-up visits. Not surprisingly, this requires significant effort with multiple phone calls, multiple stakeholders, and much time in order to ensure successful treatment initiation and compliance. Given the workload, the nurse coordinator estimates a maximum possible patient load of fifteen patients at any given point in time.

### Facilitators and barriers to HCV treatment delivery

A summary of the findings related to the facilitators and barriers of HCV treatment delivery are presented in Table 2. All members of the HCV care team noted that insurance authorization for treatment was the most resource-intensive step in HCV treatment delivery and served as the greatest barrier to initiating therapy in a timely manner, noting that the process could take at least four to six weeks and even longer in cases where appeals need to be submitted. Additionally, the team overwhelmingly focused on insurance coverage as the deciding factor on whether eight or twelve week HCV treatment was used. In fact, the physicians we interviewed noted that most patients deferred to their clinical expertise in terms of the choice of prescribed regimen, but estimated that the majority of their patients receive twelve-week therapy, primarily driven by insurance coverage. In fact, a review of the medical record data from the clinic found that following the July 2017 approval of the eight week treatment regimen (Glecaprevir/pibrentasvi) until the time of the study interviews in 2019, only 10% of the 181 patients received the shorter duration regimen. The team noted that the amount of time required for insurance approval did not vary by treatment duration. However, there was an additional time burden coordinating refill and delivery for the twelve-week regimen because most insurance companies only authorize a 30-day supply of treatment.

**Table 2:** Provider perspectives of facilitators and barriers of HCV treatment delivery among patients receiving care in a large, urban healthcare system in the United States (2019).

Domain	Factor	Description	Noted by
**Predisposing factor**	Age	No impact on treatment initiation overall; preference for shorter treatment for younger age group	Two physicians
	Sex	No impact on treatment initiation	One physician
	SES	Barrier to treatment initiation overall and duration of treatment to the extent that SES is associated with health insurance status	All interviewed
	Homelessness	Barrier to treatment initiation overall; provider preference for shorter treatment regimen given competing priorities/needs among those with unstable housing	One physician and pharmacist
	Substance use	Barrier to treatment initiation overall; provider preference for shorter treatment regimen given competing priorities/needs among those with substance use	One physician
	Trust in provider	Facilitator of treatment overall and by treatment duration; providers noted that patient trust in the provider played a significant role in initiation treatment and the choice of treatment	Two physicians
**Enabling factors**	Availability of medical services/facilities	Facilitator of treatment overall	Two physicians
	Physician diagnosis	Facilitator of treatment overall and by treatment duration; mediated by ‘trust in provider’ in that once a diagnosis was made the patient’s trust in provider/diagnosis served to facilitate treatment initiation	All physicians
	Health insurance status	Lack of health insurance was a primary barrier; type of health insurance and level of health insurance coverage was also a barrier and dictated type/duration of treatment patient could receive	All interviewed
	Income/financial situation	Lack of financial resources was a primary barrier to treatment initiation and related to health insurance status	All interviewed
	Availability of financial assistance programs	Facilitator of treatment initiation; programs such as the patient assistance programs for medications which help to defray any out of pocket costs that may not be covered by insurance	Pharmacist
	Administrative support/use of case manager	Facilitator of treatment initiation and critical in preparing insurance authorization paperwork	One physician, nurse, and pharmacy case manager
**Need**	Physician evaluated health status	Facilitator of treatment overall and by treatment duration; mediated by ‘trust in provider’ and ‘physician diagnosis’ related to the patient overall health status and other competing health needs	One physician
	Self-reported health status/competing medical needs	Barrier of treatment initiation overall; barrier to shorter treatment regimen among those who are taking other medications given the increased daily pill count with the shorter regimen (as compared to the longer regimen)	Two physicians
	Daily activities/lifestyle	Barrier of treatment initiation overall; facilitator of shorter treatment regimen in that those with busier lifestyles given a preference for shorter regimen; related to age and those who are younger having a preference for shorter treatment duration	All physicians

Beyond insurance, the medical team noted that pill burden can be a deciding factor among the minority of patients who have a choice between eight or twelve week treatment. For instance, providers felt that patients taking other medications seem to have a preference for the longer treatment regimen (Sofosbuvir/velpatasvir for twelve weeks) given the one pill per day dosing as compared to the larger daily pill burden for the shorter, eight week treatment (Glecaprevir/pibrentasvir for eight week with three pills per day). Providers also felt that age affected treatment preference, with younger patients preferring the shorter treatment regimen as compared to older patients, including ‘baby boomers,’ a generational cohort highly impacted by HCV which constitutes a large proportion of patients receiving HCV care [32]. The treatment team also noted unique situations in which shorter treatment duration is preferred. For instance, the physicians noted a preference for shorter treatment regimen for patients that have been historically difficult to maintain in regular care, such as those who are marginally housed or those with substance use issues who may have challenges with compliance. The providers noted that if they are able to identify a period of time in which the patient is likely to be more compliant with treatment, then there is a strong preference for shorter treatment in order to increase uptake and adherence. Likewise, the pharmacist noted that the blister packs used for the eight week treatment regimen (as compared to prescription vials) were particularly well suited for those who may have compliance issues. However, those considerations were only secondary to insurance coverage.

## Discussion

Overall we found that the process of insurance authorization for HCV treatment—even in the context of a well-resourced health system—to be laborious and serve as one of the greatest obstacles to initiation of shorter HCV treatment regimen. We also found that a model of care involving close collaboration between a full team of providers is key to the success of treatment delivery, which has allowed for linkage to care and functional cure in 94% of HCV patients within this health system [33]. In an era with access to therapies that allow for a cure, implementing strategies that can expedite treatment access will not only benefit patients but will help expand treatment delivery to resource strained health care settings which may not otherwise be able to take on the administrative work load associated with HCV treatment initiation. Additionally, by reforming our approach to allow for insurance coverage of shorter duration treatment, we can expand resources available for treatment delivery and increase the number of HCV infected persons treated, aligning more closely with both national and international targets to increase HCV treatment and reduce the number of chronic HCV infections [28, 29].

In 2020 the US Preventative Task Force updated its HCV screening guidelines from a ‘risk-based’ screening approach to recommend one-time screening for HCV infection for adults 18 to 79 years, regardless of risk [34, 35]. This suggests not only an increase in screening but a resulting increase in therapeutic demand. Several features of the current HCV treatment paradigm in the United States makes achieving these targets and meeting potential increases in treatment demand challenging and strategies to streamline treatment delivery are critical. First, the task of submitting prior authorization requests to insurance companies, appealing denials, and coordinating with patients and the extended medical care team can delay treatment, increase loss to follow-up, and serve as an additional resource burden on an already strained system. A study conducted by the American Medical Association found that among the 1,000 primary and specialty care physicians surveyed, an average of two business days per week was devoted to prior authorization requests and the overwhelming majority (91%) felt that the prior authorization process had a negative impact on clinical outcomes [36]. Our qualitative data confirm these findings and provide further insight to the specific context of HCV treatment, highlighting the fact that dedicated staff including clinical and pharmacy staff are critical to ushering the pre-authorization process through successfully and thus limiting HCV treatment to well-resourced health care settings.

Prior authorization for medications were initially put into place to address higher cost prescriptions that may have lacked medical justification and directed to newer high cost drugs with limited benefit [37]. When DAAs first became available, widespread treatment was cost-prohibitive—given the $1,000 per pill price tag—and treatment was limited to those with advanced liver disease who were likely to have the best outcome [24]. As generic treatment regimen have become available and treatment duration has decreased, it’s estimated that the cost of curing HCV is less than the annual cost of HIV treatment (which does not require prior authorization for treatment) [38]. Elimination of prior authorization for HCV treatment seems particularly relevant given that in most cases the insurance denial rate is low, treatment can successfully lead to cure in the majority of cases, costs have declined in recent years, and alternative treatment which are lower in cost are currently not available [17, 24, 39, 40].

Until the revocation of pre-authorization for HCV treatment is a reality, strategies that improve efficiency in the process are critical. Introducing non-clinical staff to serve as insurance authorization coordinators can help decrease nursing staff workload. Task-shifting administrative roles has the potential to free resources, allowing for increased sustainability of a program that is heavily reliant on a treatment coordinator. That task-shifting may also allow scaling of HCV treatment from specialty clinics into other settings such as primary care, where providers may not have nursing time available to devote to the process. Second, access to treatment may be increased by expansion of insurance coverage to allow for a choice of treatment (i.e., eight or twelve week regimen), especially in cases where a shorter treatment regimen has the chance of improving compliance such as those who are unstably housed or have competing substance use issues. Additionally, ensuring prompt and continuous access to medications by eliminating the need for refills by dispensing the full course of treatment when possible can help streamline the process from both the provider and patient perspective.

Several limitations of this study should be noted. First, the sample size is relatively small, therefore additional barriers and facilitators of HCV care not mentioned by the providers we interviewed would not be detected. However, the primary theme around the resource intensive nature of the prior authorization process was repeated by all interviewees which allowed for “saturation” around this theme. Second, the findings were in the context of a well-resourced large healthcare system and may not be relevant to other health settings. Nonetheless, our results demonstrate that despite improvements in treatment for HCV, much work remains for improving the continuum of HCV care. Changes in policies around prior authorization for curative HCV treatment have the potential to increase treatment capacity allowing us to get one step closer to the treatment goal of 80% of eligible patients, ultimately helping to accelerate the drive toward HCV elimination targets [41].
